# A Preliminary Exploration of Former Smokers Enrolled in an Internet Smoking Cessation Program

**DOI:** 10.2196/resprot.5542

**Published:** 2016-06-14

**Authors:** Sarah Cha, Amy M Cohn, Hoda Elmasry, Amanda L Graham

**Affiliations:** ^1^ Schroeder Institute for Tobacco Research and Policy Studies at Truth Initiative Washington, DC United States; ^2^ Department of Oncology Georgetown University Medical Center / Cancer Prevention and Control Program Lombardi Comprehensive Cancer Center Washington, DC United States

**Keywords:** smoking cessation, Internet, relapse prevention

## Abstract

**Background:**

Internet interventions may have an important role to play in helping self-quitters maintain an initial period of abstinence. Little is known about the characteristics and utilization patterns of former smokers who use Internet cessation programs.

**Objective:**

The overarching aim of this preliminary study was to establish the feasibility of a subsequent randomized trial of the effectiveness of Internet interventions in preventing relapse. Specifically, this study sought to determine the number of former smokers that register on a smoking cessation website, the characteristics of former smokers and their website utilization patterns, and potential predictors of sustained abstinence.

**Methods:**

Participants were self-identified former smokers who registered on a free smoking cessation website. Recruitment occurred immediately following site registration. Participants completed Web-based baseline and 1-month follow-up assessments. Website utilization metrics were extracted at 1 month. Descriptive statistics were used to characterize the full sample. Baseline differences were examined between recent quitters (≤7 days of abstinence at enrollment) and more established quitters (8+ days of abstinence at enrollment) using chi-square tests and *t* tests. Univariate logistic regression examined demographic, smoking, psychosocial characteristics, and website utilization metrics as predictors of 1-month abstinence.

**Results:**

During the 10-month study period, 1141 former smokers were recruited to participate: 494 accepted the invitation, 395 were eligible, 377 provided informed consent, and 221 completed the baseline and fully enrolled (56% of those eligible). At 1 month, 55.7% (123/221) of participants completed the follow-up survey. Mean age was 44.25 years (SD 12.78) and the sample was primarily female (174/221, 78.7%), white (196/221, 88.7%), and had at least some college education (177/221, 80.1%). Slightly more than half of participants (123/221, 55.7%) reported quitting more than a week prior to website registration and 43.9% (97/221) had quit within 7 days of registration. The website features most likely to be used were an interactive Quit Date tool (166/221, 75.1%) and the Community (134/221, 60.6%). Univariate regression models showed that recent quitters, those with higher motivation to remain abstinent, and those who used cessation medication in the past year were more likely to use the Community. Older age, longer duration of abstinence at registration, better health status, and health care provider advice to quit were associated with 1-month abstinence. Website utilization metrics did not predict abstinence, though odds ratios suggested higher utilization was associated with greater odds of abstinence.

**Conclusions:**

This exploratory study demonstrated the feasibility of recruiting former smokers to a research study and documented the uptake of an Internet cessation intervention among this group of self-quitters. Results also showed higher levels of website utilization and greater likelihood of community use among smokers early in their quit attempt compared to those with a longer period of abstinence at enrollment. Important areas for future research include identifying former smokers who may be more susceptible to relapse and determining which components of an Internet intervention are most helpful to prevent relapse in the early and later stages of a quit attempt.

## Introduction

Identifying strategies to prevent relapse among the millions of smokers that attempt to quit each year remains a public health priority [1]. In 2012, 52.9% of smokers attempted to quit smoking, yet each year, only 5-7% of smokers are able to maintain abstinence for more than 6 months [2]. The majority of smokers relapse within several weeks after a quit attempt [3-6]. Despite decades of research on relapse prevention, a 2013 Cochrane systematic review of 63 studies found little support for the effectiveness of behavioral interventions delivered face-to-face or telephonically, or for pharmacological interventions [7]. As tobacco control policies continue to drive increased quit attempts [8], novel relapse prevention efforts are needed to increase the likelihood that these efforts translate into successful long-term cessation [9].

Internet interventions may be uniquely suited to provide relapse prevention approaches for smoking cessation as they are broad-reaching [10,11], cost-effective [12], and may appeal to individuals who would otherwise not seek cessation counseling. The Internet is a primary source of health information for a majority of adults [13] and is often the first place many people turn to when faced with a health-related question or concern [14]. A timely, on-demand intervention after a smoking lapse is a critical element of an effective relapse prevention approach [15] and one that other treatment modalities like face-to-face and telephonic interventions may be unable to provide. The 24/7 availability of Internet interventions and their ability to surmount geographic and other barriers to treatment use make them a powerful channel through which to address relapse at the time when support is most needed.

In addition, Internet interventions for smoking cessation commonly include social media and Web 2.0 applications that facilitate the exchange of information and support between and among users [16]. Real-time social support from current and former smokers may provide precisely the kind of encouragement, inspiration, and “road-tested” practical advice that former smokers need to prevent relapse [17]. High levels of social support have been associated with better cessation outcomes in a number of studies [18-20], and low levels of support have been conceptualized as a barrier to abstinence [21]. Few studies have examined the role of “offline” social support for relapse prevention [7], and only two studies to our knowledge have explored the impact of online social support through Internet cessation interventions. Schwarzer and Satow [22] found that recent quitters who posted the number of days they had been abstinent in an online bulletin board were less likely to relapse than those who did not post. In addition, posting more messages was associated with a greater likelihood of maintaining abstinence. The authors posited that making one’s intentions to quit visible to an online community may strengthen an individual’s commitment to quit and that active engagement naturally results in continued contact with others, being reminded of one’s intention to quit, and potentially receiving praise. Selby et al [17] found that among individuals who posted within an online cessation community, the most common type of first posts were help-seeking messages from recent quitters who were struggling to remain abstinent. In this study, we were specifically interested in exploring whether use of an online community for smoking cessation was associated with lower rates of relapse among former smokers.

Internet interventions have shown promise for relapse prevention in mental health and addiction treatment [23,24], and several studies within smoking cessation suggest that there is demand for and utilization of online cessation resources among recent quitters. A study by Borland et al [25] sought to examine the impact of a Web-based intervention among current smokers and recent quitters (defined as quit for <4 days) but did not report outcomes by baseline smoking status. Interestingly, approximately 25% of participants screened for this smoking cessation study reported they had already quit smoking. Similarly, a 2006 study by Cobb and Graham [10] found that 24% of individuals searching for smoking cessation information on the Internet had quit smoking: 17% had quit within the previous 7 days, and 7% had quit more than 7 days prior. In an observational study of the Australian Web-based cessation program “QuitCoach,” Balmford et al [26] found that return visits were most common among those who had just quit when they registered on the site and lowest among those not planning to quit.

These studies and others [11,27,28] suggest that Internet interventions may have an important role to play in helping those who have already quit to maintain an initial period of abstinence or to extend their abstinence. However, to date, few studies have documented the extent to which former smokers use Internet cessation programs or their effectiveness in preventing relapse. The goals of this preliminary study were to explore the feasibility of conducting a randomized trial to evaluate the effectiveness of Internet interventions in preventing relapse. Specifically, we sought to address the following questions: (1) Is it feasible to recruit former smokers to a research study, and what is the monthly recruitment volume?, (2) What are the characteristics of former smokers that register on an Internet smoking cessation website?, (3) How do former smokers utilize an Internet cessation program?, and (4) Are there baseline variables or website utilization metrics that predict a former smoker’s ability to maintain abstinence?

## Methods

### Participants

Participants were individuals who registered on a free smoking cessation website and selected “former smoker” when asked about smoking status (options were “current smoker,” “former smoker,” or “looking for help for someone else”). The only other eligibility criteria were age 18 years or older and US residence, which were gathered during website registration. The study invitation was presented immediately following website registration. Eligibility screening, informed consent, and the baseline survey were administered online. Immediately following the baseline survey, participants were directed back to the website where they were able to use the site as they desired; there were no additional interventions provided. At 1-month post-registration, participants were asked to complete an online survey to assess smoking status and other related variables. Participants received three email prompts for follow-up survey completion and were offered a US $20 incentive. The study protocol received human subjects protection approval from Copernicus Group Independent Review Board.

### Intervention

BecomeAnEX is a free smoking cessation website developed and managed by Truth Initiative (formerly American Legacy Foundation) in partnership with the Mayo Clinic Nicotine Dependence Center [11,29]. Consistent with the 2008 Public Health Service Clinical Practice Guideline for Treating Tobacco Dependence [1], BecomeAnEX provides (1) problem-solving and skills training designed to enhance self-efficacy, (2) information and guidance in selecting and using FDA-approved smoking cessation pharmacotherapies, and (3) intra-treatment social support in the form of a large online community. BecomeAnEX guides and supports smokers through the process of planning and preparing to quit through the following interactive features: (1) a Quit Date tool that assists users in selecting a prospective quit date or documenting a retrospective quit date, (2) a Cigarette Tracker exercise to identify smoking triggers, (3) a Beat Your Smoking Triggers exercise (Separation exercise) to identify strategies to dissociate cigarettes from triggers, (4) a Build Your Support System exercise (Support exercise) to identify helpful supporters, (5) a Choose a Quit Smoking Aid exercise (Addiction exercise), in which users indicate their plans for pharmacotherapy use, and (6) Community, which is a large online network of thousands of current and former smokers who communicate through a variety of channels (eg, blog posts/replies, wall posts, private messages). In addition to these interactive features, the site contains static content to prepare for quit day, cope with slips, and prevent relapse; videos about addiction and medication; and a checklist (My Quit Plan) that displays whether each of the site’s core components has been used and recommends next steps. The site can be browsed anonymously, but to save information or post content in the Community, visitors must register. To register on BecomeAnEX, individuals must agree to the site’s Terms of Use and Privacy Policy. The Privacy Policy makes clear that (1) BecomeAnEX automatically collects information about its users and their use of the site, (2) information is used for research and quality improvement purposes only, and (3) personal information is kept confidential. BecomeAnEX has been promoted through a national multimedia campaign since 2008 [11], with more recent promotional activities focused on paid search advertising that targets current smokers.

### Data Collection and Measures

Data sources for these analyses included (1) a Web-based survey administered at baseline, (2) a Web-based survey administered at 1-month post enrollment, and (3) 1-month website utilization metrics obtained via automated tracking software. Demographic variables included age, gender, race, education, employment, and marital status.

Abstinence-related questions asked when participants decided to quit smoking, their quit date and confidence about the accuracy of that date, and the date of their last puff of a cigarette. These questions were used to calculate the number of days they had been abstinent when they registered on BecomeAnEX. The Abstinence Related Motivational Engagement Short Form (ARME) [30] was administered to assess motivation to remain abstinent. This scale consists of 5 Likert items (1=completely disagree to 7=completely agree): (1) I try to anticipate and prepare for any challenges to being smoke-free (vigilance), (2) The thought of being a nonsmoker still excites me (excitement), (3) At this time, I am still very excited by the idea of being smoke-free (excitement), (4) I spend a great deal of time thinking about becoming or staying smoke-free (cognitive effort), and (5) I am carefully watching out for things that might put me at risk for smoking (vigilance). The short form has demonstrated adequate reliability (alpha=.82) and has been correlated with length of abstinence [31].

Smoking history questions asked about the number of quit attempts made in the past year, and the use of behavioral (books/pamphlets, individual/group counseling, telephone quitline, Web-based interventions), pharmacologic (nicotine patches, gum, lozenges, nasal spray and inhaler, Zyban, Chantix), and alternative (e-cigarettes, switching to chew or snuff, switching brand or cutting back, acupuncture, hypnosis, herbal/laser/other alternative methods) quit methods during the past year. Reports of using “willpower/cold turkey” or “prayer” were coded as unassisted quit attempts.

Health history items included current health status (excellent, very good, good, fair, poor; [32]), history of an illness caused or made worse by smoking (yes/no), and whether the participant had received advice to quit smoking from a health care provider in the past year (yes/no).

Psychosocial measures assessed “offline” social support as potential influences on online Community use. They included the Appraisal and Belonging subscales of the 12-item Interpersonal Support Evaluation List [33], which is a general measure of perceived social support. The Appraisal subscale measures the perceived availability of someone to talk to about one’s problems, and the Belonging subscale measures the perceived availability of people one can do things with. Each subscale contains four statements that participants indicate are definitely true, probably true, probably false, or definitely false. One item from the UCLA Loneliness Scale [34] was administered, which asked how often the participants felt that there were people they can turn to (1=Never, 4=Always).

Internet and social media use were assessed with items that asked about frequency and duration of Internet use [35] and frequency of communication with other people via the Internet (eg, via blogs, instant messaging, forums) [36].

Website utilization data were obtained via Adobe Analytics software [37], a customizable Web analytics tool that is used to monitor, report on, and optimize use of the BecomeAnEX website. General utilization metrics examined in this study included number of return visits following website registration, total number of minutes spent on the site, and the number of pages viewed. Data were also extracted on the use of the six interactive features described above. Because website utilization typically shows a steep attrition curve [38-43], we focused on utilization metrics during the first month following site registration.

Smoking abstinence at 1-month post enrollment was measured as 7-day and 30-day point prevalence abstinence.

### Statistical Analyses

Descriptive statistics were examined to characterize former smokers on sociodemographic variables, smoking history, health status, and psychosocial measures. We also examined baseline differences between recent quitters (≤7 days of abstinence at enrollment) and more established quitters (8+ days of abstinence at enrollment) using chi-square tests and *t* tests. Our decision to use 7 days as a cut-point was largely an empirical one. Relapse is most common within the first week after a quit attempt, and prior analyses have shown that the largest proportion of Internet cessation treatment users that self-identify as former smokers report quitting within the past 7 days [3,10,44]. Previous studies of relapse prevention interventions have varied widely in terms of abstinence-related inclusion criteria [45]. Our intent was to determine if there were distinguishing characteristics based on length of abstinence at program enrollment that might suggest that a subsequent effectiveness study should focus specifically on recent quitters.

Website utilization patterns were examined using descriptive statistics. The full sample was characterized, and comparisons between recent quitters and more established quitters were explored. Means and standard deviations were computed for general website utilization variables and compared using two-sample *t* tests. Given that general website utilization data were positively skewed, the median and interquartile range are also reported and differences examined using the Wilcoxon Mann Whitney test.

To identify characteristics of participants who used the Community, univariate logistic regression models examined baseline demographic, smoking history, and psychosocial variables. To identify predictors of 1-month abstinence, univariate logistic regression models examined the association between 30-day abstinence and baseline characteristics (demographic, smoking history, psychosocial measures), website utilization metrics (return visits, time on site, Community use), and other treatment utilization (behavioral interventions, medication use, alternative methods). Statistical significance for all analyses was set to an alpha of .05. Analyses were performed using SPSS version 21 and SAS software version 9.3.

## Results

### Recruitment and Follow-Up Results

Between November 15, 2012, and September 17, 2013, a total of 1141 consecutive registered users who identified as former smokers were recruited to participate in the study: 494 accepted the invitation, 395 were eligible, 377 provided informed consent, and 221 completed the baseline survey and fully enrolled (56% of those eligible). This represents an available pool of approximately 114 former smokers per month from which to recruit and a recruitment rate of approximately 22 participants per month. At 1-month post registration, 55.7% (123/221) of participants completed the follow-up survey. Survey non-respondents were more likely to have a high school degree or less (OR 2.68, 95% CI 1.35-5.30) and to be black/African American (OR 4.26, CI 1.14-15.97). One-month follow-up attrition was significantly correlated with website utilization. Specifically, those with fewer site visits, time on site, and number of page views were also more likely to be lost to follow-up (all ORs ˃1.76, *P*<.04).

### Baseline Characteristics of Former Smokers

Table 1 shows the demographic, smoking history, and psychosocial characteristics of the full sample of former smokers. Mean age was 44.25 years (SD 12.78), and the sample was primarily female (174/221, 78.7%), white (196/221, 88.7%), college educated (177/221, 80.1% reporting some college or more), employed full or part-time (149/221, 67.4%), and married or living with a partner (135/221, 61.1%). Two thirds (148/221, 67.0%) reported having an illness either caused or made worse by smoking, and 69.7% (154/221) had been advised to quit by a health care provider in the past year. The average score for ARME was high (mean 29.51, SD 5.69), with the two “excitement” items (“thought of being nonsmoker still excites me,” “I am still very excited by the idea of being smoke-free”) yielding the highest mean values (mean 6.1, SD 1.5 for both items).

**Table 1:** Baseline characteristics of former smokers by number of days quit at enrollment.

			All former smokers, N=221	Days quit at enrollment		
				≤7 days, n=97	8+ days, n=123	*P* value^a^
**Demographic characteristics**						
	Age, years, mean (SD)		44.25 (12.78)	40.39 (11.82)	47.33 (12.76)	<.001
	Gender, female, n (%)		174 (78.7)	74 (76.3)	99 (80.5)	.45
	Race, white, n (%)		196 (88.7)	85 (87.6)	110 (89.4)	.76
	Ethnicity, Hispanic, n (%)		12 (5.4)	6 (6.2)	6 (4.9)	.67
	Education, some college or more, n (%)		177 (80.1)	76 (78.4)	100 (81.3)	.59
	Employment, full-time or part-time, n (%)		149 (67.4)	67 (69.1)	82 (66.7)	.70
	Marital status, married/partner, n (%)		135 (61.1)	54 (55.7)	81 (65.9)	.12
**Smoking history**						
	ARME, range (5-35), mean (SD)		29.51 (5.69)	30.46 (4.89)	28.75 (6.18)	.03
	# quit attempts past year, mean (SD)^b^		2.29 (2.90)	2.21 (2.73)	2.34 (3.04)	.74
	Quit methods, #quit attempt past year ≥1, n (%)		N=182	N=74	N=107	
		Unassisted	131 (72.0)	51 (68.9)	79 (73.8)	.47
		Behavioral interventions	58 (31.9)	22 (29.7)	36 (33.6)	.58
		Medications	135 (74.2)	56 (75.7)	78 (72.9)	.68
		Alternative methods	81 (46.6)	34 (46.6)	46 (46.0)	.94
**Health status, n (%)**						
	Self-reported health status, fair or poor		50 (22.6)	18 (18.6)	31 (25.2)	.24
	History of smoking-related illness		148 (67.0)	61 (62.9)	86 (69.9)	.27
	Health care provider advice to quit past year		154 (69.7)	66 (68.0)	87 (70.7)	.72
**Psychosocial variables**						
	ISEL^c^ Appraisal subscale, range (1-12), mean (SD)		7.63 (2.03)	7.82 (1.85)	7.48 (2.16)	.21
	ISEL Belonging subscale, range (1-12), mean (SD)		7.02 (1.39)	7.12 (1.26)	6.95 (1.49)	.36
	Loneliness, never or rarely, n (%)		19 (8.6)	10 (10.3)	9 (7.3)	.43
**Internet use, n (%)**						
	How long used Internet, ˃5 years		208 (94.1)	91 (93.8)	116 (94.3)	.80
	How often use Internet, several times/day		176 (79.6)	81 (83.5)	94 (76.4)	.34
	Use of Internet to blog/chat/instant message					.74
		Several times/day	80 (36.2)	38 (39.2)	42 (34.1)	
		Once a day	42 (19.0)	18 (18.6)	24 (19.5)	
		Less than daily	99 (44.8)	41 (42.3)	57 (46.3)	

^a^Former smokers who had quit within the past 7 days at enrollment compared to former smokers who had quit 8 days or more at enrollment. One respondent who did not provide valid date-based responses was excluded from comparison but included in all former smokers column.

^b^Quit attempts were restricted to ≤20 attempts, removing 1 outlier.

^c^ISEL=Interpersonal Support Evaluation Scale.

**Table 2:** One-month website utilization metrics of former smokers by days quit at enrollment.

		All former smokers, N=221	Days quit at enrollment
			≤7 days, n=97	8+ days, n=123	*P* value^a^
**General website utilization**					
	No. return visits, mean (SD)	6.61 (17.79)	8.79 (21.46)	4.93 (14.19)	.13
	No. return visits, median (IQR)	2.00 (1.00-4.00)	3.00 (2.00-6.00)	2.00 (1.00-3.00)	.01
	Time on site (minutes), mean (SD)	115.69 (433.42)	159.60 (549.90)	81.93 (313.10)	.22
	Time on site (minutes), median (IQR)	28.27 (14.50-56.40)	42.33 (18.30-75.90)	25.37 (11.90-39.50)	.02
	No. page views, mean (SD)	82.00 (228.33)	110.70 (279.90)	59.98 (176.60)	.12
	No. page views, median (IQR)	32.00 (11.00-58.00)	39.00 (18.00-88.00)	26.00 (9.00-45.00)	.05
**Feature utilization, n (%)**					
	Set a Quit Date	166 (75.1)	85 (87.6)	81 (65.9)	<.001
	Visited Community	134 (60.6)	68 (70.1)	66 (53.7)	.01
	Choose a quit smoking aid	63 (28.5)	34 (35.1)	29 (23.6)	.06
	Separation exercise	62 (28.1)	33 (34.0)	29 (23.6)	.09
	Addiction videos	102 (46.2)	52 (53.6)	50 (40.7)	.06
	Support exercise	38 (17.2)	20 (20.6)	18 (14.6)	.24
	Cigarette tracker	34 (15.4)	13 (13.4)	20 (16.3)	.56
**Community utilization, n (%)**					
	Viewed user profiles	36 (16.3)	20 (20.6)	16 (13.0)	.13
	Read blog posts	29 (13.1)	19 (19.6)	10 (8.1)	.01
	Wrote blog posts	19 (8.6)	13 (13.4)	6 (4.9)	.03
	Wrote on user message board	13 (5.9)	10 (10.3)	3 (2.4)	.01
	Sent private messages	11 (5.0)	8 (8.3)	3 (2.4)	.05

^a^Recent quitters (≤7 days abstinence at enrollment) compared to more established quitters (8+ days abstinence at enrollment). One respondent who did not provide valid date-based responses was excluded from comparison but included in all former smokers column.

**Table 3:** Univariate logistic regression model of odds of 30-day abstinence at 1 month among former smokers.

Variable		Group	Abstinent, n=83	Smoking, n=40	Crude OR	95% CI	*P* value
**Demographic variables**							
	Age (5-year increments)				1.23	1.05-1.40	.01
	Gender						
		Female (ref)	66	30	—	—	
		Male	17	10	0.77	0.32-1.89	.57
	Education						
		HS or less (ref)	9	7	—	—	
		Some college or more	74	33	1.74	0.60-5.08	.31
	Race						
		White (ref)	74	36	—	—	
		Non-white	9	4	1.10	0.32-3.80	.89
	Ethnicity						
		Non-Hispanic (ref)	79	37	—	—	
		Hispanic or Latino	4	3	0.62	0.13-2.93	.55
	Employment status						
		Not employed (ref)	22	14	—	—	
		Employed	61	26	1.49	0.66-3.36	.33
	Marital status						
		No partner (ref)	29	19	—	—	
		Partner	54	21	1.69	0.78-3.63	.18
**Smoking variables**							
	Consider self former smoker						
		Within past week (ref)	33	29	—	—	
		More than a week ago	42	8	4.61	1.87-11.41	<.001
	Days quit at enrollment						
		8+ days (ref)	55	10	—	—	
		≤7 days	28	30	0.17	0.07-0.40	<.001
	ARME				1.01	0.94-1.09	.70
**Past year quit methods**							
	Unassisted						
		No (ref)	17	9	—	—	
		Yes	56	21	1.41	0.55-3.65	.48
	Behavioral						
		No (ref)	47	21	—	—	
		Yes	26	9	1.29	0.52-3.23	.59
	Medication						
		No (ref)	16	9	—	—	
		Yes	57	21	1.53	0.59-3.98	.39
	Alternative methods						
		No (ref)	39	14	—	—	
		Yes	28	16	0.63	0.26-1.49	.29

**Health status**							
	Health						
		Fair/poor (ref)	13	13	—	—	
		Excel/very good/ good	70	27	2.59	1.07-6.30	.04
	Illness from smoking						
		No (ref)	24	16	—	—	
		Yes	59	24	1.64	0.74-3.61	.22
	Health care provider advice to quit						
		No (ref)	19	16	—	—	
		Yes	64	24	2.25	1.00-5.07	.05
	Communicate via Internet						
		Less than daily (ref)	62	37	—	—	
		Daily or more often	68	54	0.80	0.38-1.70	.56
**Past month quit methods at follow-up**							
	Unassisted						
		No (ref)	21	13	—	—	
		Yes	62	27	1.42	0.62-3.25	.40
	Behavioral interventions						
		No (ref)	47	25	—	—	
		Yes	36	15	1.28	0.60-2.77	.54
	Medication						
		No (ref)	45	21	—	—	
		Yes	38	19	0.93	0.44-1.99	.86
	Alternative methods						
		No (ref)	51	24	—	—	
		Yes	32	16	0.94	0.44-2.04	.88
**1 month website utilization**							
	2+ return visits						
		No (ref)	14	12	—	—	
		Yes	69	28	2.11	0.87-5.13	.10
	30+ minutes on site						
		No (ref)	37	20	—	—	
		Yes	46	20	1.24	0.58-2.65	.57
	2+ community visits						
		No (ref)	29	19	—	—	
		Yes	54	21	1.69	0.78-3.63	.18

Slightly more than half of participants (123/221, 55.7%) were “more established quitters” (days abstinent at registration: mean 358.8, SD 1504.9, range 9713) and 43.9% (97/221) of the sample were “recent quitters” (days abstinent at registration: mean 3.1, SD 2.0, range 7). One respondent did not provide valid date-based quitting-related responses and the length of their quit at registration is unknown. Recent quitters were younger (mean 40.39, SD 11.82 vs mean 47.33, SD 12.76, *P*<.001) and had higher scores on the ARME (mean 30.46, SD 4.89 vs mean 28.75, SD 6.18, *P*=.03) than more established quitters. No other baseline differences were observed.

### One-Month Website Utilization Patterns and Predictors of Community Use

Website utilization metrics are presented in Table 2. During the first month after registration, participants made an average of 6.61 return visits to the site (SD 17.79; median 2.00), spent 115.69 minutes on the site (SD 433.42; median 28.27), and viewed 82.00 pages (SD 228.33; median 32.00). The most commonly used features were Set a Quit Date (75.1%) and Community (60.6%). There were significant differences between recent quitters and more established quitters across a number of utilization metrics. Recent quitters made more return visits (median 3.00, interquartile range (IQR) 2.00-6.00 vs median 2.00, IQR 1.00-3.00, *P*=.01), spent more time on the site (median 42.33, IQR 18.30-75.90 vs median 25.37, IQR 11.90-39.50, *P*=.02), and viewed more pages (median 39.00, IQR 18.00-88.00 vs median 26.00, IQR 9.00-45.00, *P*=.05), compared to more established quitters. Recent quitters were also more likely than more established quitters to set a quit date on the site (87.6% vs 65.9%, *P*<.001), to visit the Community (70.1% vs 53.7%, *P*=.01), and to engage in the Community both passively (read blog posts: 19.6% vs 8.1%, *P*=0.01) and actively (wrote a blog post: 13.4% vs 4.9%, *P*=.03; wrote on message boards: 10.3% vs 2.4%, *P*=.01; sent private messages: 8.3% vs 2.4%, *P*=.05).

### Predictors of Abstinence

Univariate regression analyses showed that several baseline characteristics were predictive of 1-month abstinence (see Table 3). Older age (OR 1.23, CI 1.05-1.40), self-identification as a more established quitter (OR 4.61, CI 1.87-11.41), better health status (OR 2.59, CI 1.07-6.30), and being advised by a health care provider to quit in the past year (OR 2.25, CI 1.00-5.07) were associated with increased abstinence, whereas 7 or fewer days of abstinence at registration was associated with lower odds of 1-month sustained abstinence (OR 0.17, CI 0.07-0.40). General website utilization metrics (number of return visits, time on site) and community use did not emerge as significant predictors of abstinence, though odds ratios suggested that higher levels of utilization were associated with increased abstinence.

## Discussion

### Principal Findings

This study is one of the first to characterize a sample of former smokers that registered on an evidence-based Internet smoking cessation program, document their website utilization patterns, and explore the factors that predicted maintenance of an initial period of abstinence. Over the 10-month study period, 1141 former smokers registered on the site. This is noteworthy given that all promotional efforts describe the site as a smoking cessation intervention for current smokers. Promotional efforts that specifically appeal to recent quitters may attract an even larger audience, as our data demonstrate that an online cessation program is of interest to recent quitters looking for information and support. The study enrollment rate is comparable to several recent large-scale Internet cessation trials [25,46] and demonstrates the feasibility of recruiting former smokers to participate in research.

In general, this was a sample of very recent quitters, nearly half of whom had quit within the past week and who were very motivated to maintain this initial period of abstinence. Two-thirds reported having an illness caused or made worse by smoking and having been advised by a health care provider to quit smoking. Participants had made multiple quit attempts in the past year, and the majority had used medication and alternative quit methods during these quit attempts. These characteristics paint a picture of middle-aged smokers who had experienced multiple failed quit attempts using other treatment strategies but who were still engaged in the process of quitting. That the sample was largely female is consistent with reports that women are more likely than men to seek health care information online [13].

Self-identified former smokers were not a homogeneous group when it came to website utilization patterns. Recent quitters (ie, those who had quit in the last week) returned to the site more often, viewed more pages, and spent more time on the site than more established quitters (ie, those who had quit more than a week ago). They were also more likely to use the quit date feature and to participate in the Community both actively and passively. These differences may signal the more precarious nature of their abstinence and the need for different type of guidance and support than those who are more established in their quit. It is noteworthy that of all the website features examined, the most consistent patterns of differences emerged in use of the online Community. Additional research to understand the nature of the posts that former smokers make in blogs and on message boards may help inform more tailored treatment strategies specifically designed for recent quitters versus more established quitters.

Approximately a third of participants who completed the follow-up survey indicated that they had returned to smoking at 1 month. Older age, longer duration of abstinence at enrollment, better health status, and having received advice from a health care provider to quit smoking were predictive of abstinence. These findings suggest that it may be possible to identify former smokers at higher risk for relapse using baseline characteristics, which is consistent with previous research [47]. More intensive or directed intervention for former smokers at greater risk of relapse—potentially leveraging the constant availability of online community support—may be a fruitful line of inquiry for future research.

### Limitations

This study has several limitations. First, the wide confidence intervals in several of the univariate analyses point to small cell counts for several variables. This was a feasibility study primarily designed to determine the available pool of participants for a subsequent trial and to characterize this understudied group of website users. Future research with a larger sample is needed to confirm some of the preliminary associations we have identified. Second, given the exploratory nature of the study, we did not control for the number of statistical analyses conducted so as not to miss important potential relationships. This approach may have increased the likelihood of Type I error. Third, as this was an exploratory study, univariate logistic regression results are unadjusted and the associations noted in the results section may not persist if appropriate adjustments are made. Fourth, assessing abstinence at 1-month post registration provides only an early peek at the potential effectiveness of an Internet intervention in preventing relapse. Studies with a longer-term follow-up are needed to assess the extent to which the early signals of intervention effect are sustained over time. Finally, slightly more than half the sample was reached for follow-up. Although this degree of attrition is common in Internet-based studies [48], it may have resulted in an overestimate of the proportion of participants who were abstinent in responder-only analyses and may limit the generalizability of these findings. However, our use of automated tracking data ensured that we captured the full extent of website utilization during the study period.

### Conclusion

Our findings suggest that the Internet may be a promising delivery channel for relapse prevention intervention and highlight several important areas for future studies. Additional research should focus on identifying recent quitters who may be more susceptible to relapse and determining which specific aspects of a Web-based intervention are most helpful to recent quitters in preventing relapse. Optimizing Internet interventions to help recent quitters maintain an initial period of abstinence may yield significant benefits for reducing the prevalence of smoking.

## Abbreviations

ARME: Abstinence Related Motivational Engagement
ISEL: Interpersonal Support Evaluation Scale
